# Spiritual Leadership and Job Engagement: The Mediating Role of Emotion Regulation

**DOI:** 10.3389/fpsyg.2022.844991

**Published:** 2022-04-14

**Authors:** Yicong Huang

**Affiliations:** School of Business Administration, South China University of Technology, Guangzhou, China

**Keywords:** spiritual leadership, job engagement, emotion regulation, cognitive reappraisal, expressive suppression

## Abstract

Big data era has brought big challenge for firms in human resource management, especially in employees‘ emotion. Drawing on emotion regulation and leadership theories, this study tests the mediating roles of emotion regulation for cognitive reappraisal and expressive suppression in the relationship between spiritual leadership and job engagement. We made a field survey with 203 full-time employees to test our hypotheses. Empirical results show that the mediating effects of both cognitive reappraisal and expressive suppression in accounting for the relationship between spiritual leadership and job engagement. Our study is among the first to examine whether and why spiritual leadership predicts job engagement. Our study contributes to the literatures on job engagement and emotion regulation.

## Introduction

The competitiveness of a firm greatly depends on the job engagement of its employees (Anderson and Sun, 2017); the success of its human resource management largely relies upon employees’ willingness and emotion to engage in their own jobs (Bakker et al., 2016). Job engagement, defined as “organization members control the ego and align it with the job role” (Kahn, 1990, p. 693), has been found to be necessary for achieving efficiency and good performance at the individual level (e.g., Reimer-Kirkham et al., 2012; Bayighomog and Arasli, 2022), team level (e.g., Yuan et al., 2015; Seppälä et al., 2020), and organizational level (e.g., Yang et al., 2019a). Not only is job engagement crucial for individuals and/or firms to gain better performance, it has also been regarded as emotion regulation for the engagement (e.g., Wu and Lee, 2020). Wang L. et al. (2019) has argued that employees’ refusal to engage in the jobs threatens the development and growth of the firm, damages its market competitiveness and effectiveness. Based on the real importance of job engagement, it is necessary for scholars to elaborate the factors that influence employees’ engagement in jobs (Tabor et al., 2019).

To date, studies on job engagement generally follows the leader-member exchange approach to articulate the potential antecedents of job engagement (e.g., Coat et al., 1996). While this stream of research has called for the crucial functions that contextual factors and organizational cues that incur or stop job engagement, Current research still has the following deficiencies. *First*, although some research has investigated the effects of leadership on employees’ job engagement, some domains are still unexplored. Current research focus on the effects of transformational leadership and transactional leadership on subordinates’ behavior at work. Srivastava et al. (2006) have specifically tested the importance of empowering leadership in followers’ job engagement. The development of spiritual leadership in the workplace can not only regulate the physical and mental functions of employees, re-evaluate and experience the surrounding environment, release pressure, maintain positive emotional energy in the long term, but also generate more recognition of the organization and enterprise, thus relating with emotion and engagement behavior (Crossman, 2011). However, the mechanisms linking spiritual leadership and job engagement still remain underdeveloped. *Second*, while most research has taken the leader-member exchange approach to study job engagement, little research has taken emotion lens to examine how leaders’ spirit shapes employees’ job engagement (Sheikh et al., 2019). As we see below, it is plausible that spiritual leadership might be an important antecedent of employees’ job engagement. Kahn (1990) define spiritual leadership as “a combination of mind (internal psychological traits of individuals) and leadership (explicit behaviors that effectively motivate people to achieve organizational goals)” (p. 694). There are two major pillars to spiritual leadership: a leader can emotively affect himself and his followers. Extant research has evidenced that spiritual leadership facilitates positive and desirable emotive behavior among employees (e.g., Eisenberg et al., 2000; Grant, 2008; Lesener et al., 2020). *Third*, to the extent that spiritual leadership might predict employees’ job engagement, we do not know the paths of these influence. According to the above analysis, we know that spiritual leadership play a crucial role in employees’ emotion to engage in jobs. In short, it is not clear whether the effects of spiritual leadership on followers’ behavior (e.g., job engagement) are channeled through different emotive mechanisms. To address these limitations, we seek in the present research to explore the role of spiritual leadership in shaping individual employees’ willingness of job engagement and examine the two mediation mechanisms (based on the conceptualization of emotion regulation). We propose the emotive approach as a new line for investigating the antecedents of employees’ job engagement.

Our research contributes to both theory and practice. In the view of theory, our research is crucial to the literature on leadership style and employees’ behavior. First, we analyze whether spiritual leadership can be an important antecedent of employees’ job engagement. Although job engagement has been increasingly recognized as emotive behavior (e.g., Isen et al., 1987; Posner et al., 2005; Low and Ayoko, 2018), surprisingly, no prior work has empirically investigated job engagement *via* an emotive lens. Hence, our study is the pioneer to consider spiritual leadership as a crucial antecedent of job engagement. Second, through the analyses of emotion regulation theory, we identify emotion regulation, such as cognitive reappraisal and expressive suppression as distinct pathways through which spiritual leadership is associated with job engagement. In this study we advances theory by elaborate the roles of different emotion regulation strategies in the process of spiritual leadership. Doing so, we offer a brand-new perspective for job engagement literature. From a practical perspective, our study strengthens the key function that leaders play in fostering job engagement in the workplace, thus leading to better performance and effectiveness. Leaders’ spiritual behavior will have a positive effect on employees’ engagement actions in organizations, which has major implications for firms to promote human resource management. Besides, emotion regulation strategies can adjust employee’s cognition and behavior to abide by the rule in workplace, so employees should regulate their own emotions when facing emotive crisis.

## Theory and Hypotheses

### Spiritual Leadership and Job Engagement

As shown above, job engagement refers to “the harnessing of organization members‘ selves to their work roles” (Kahn, 1990, p. 694). According to this concept, if an employee has a high level of professional engagement, he or she will be more integrated into the role behavior at work, and at the same time express himself or herself at work, and then become engaged, satisfied and passionate about his or her work.

There are two theoretical bases of job engagement, one is role theory and the other is resource conservation theory. Role theory discusses why individuals have different degrees of engagement in their workplace. A high degree of engagement in the workplace is called personal dedication to work, while a low degree of engagement is called personal alienation (Kahn, 1990). Resource conservation theory is first proposed by Hobfoll (1989) and is often used to explain the relationship between job stress and job burnout. Job requirements are regarded as the influencing factor of the threat of loss of precious resources, which is the main cause of job burnout, while job resources are regarded as the influencing factor of the acquisition of additional precious resources, which can alleviate job burnout and even improve employees‘ job engagement. Lack of work resources leads to individual alienation (Schaufeli and Bakker, 2004; Joormann and Gotlib, 2010). Job engagement is a positive, aspirational, job-related state of mind. In other words, job engagement is not a transient and specific state, but rather a persistent and emotionally permeable cognitive state that does not focus on any specific object, event, person or behavior. Individuals with high engagement and contribution have the characteristics of being energetic, and accompanied by personal enthusiasm, can effectively enter the work state and get along well with others, and feel that they are fully competent for various requirements of work (Reave, 2005).

To date, studies examining the antecedents of job engagement generally follows the leader-member exchange perspective (Coat et al., 1996). While it is a novel perspective to clarify the relationship between spiritual leadership and job engagement from the emotional view.

Spiritual leadership is a new topic in leadership research and a possible trend in the future. Some scholars believe that in the future digital economy era, new organizations must take into account both physical and spiritual functions in order to be sustainable (Shin and Hur, 2020). Otherwise, they will be easily eliminated by the trend. Spiritual leadership includes the following three elements: the first is on vision, which describes the future of the organization, defines self-positioning and actions, attaches importance to the process of vision formulation, and hopes to construct the vision of calling on employees to feel meaningful (Fry, 2003). The second is hope and confidence, that is, leaders should show a certain attitude that the vision is achievable, and believe that the mission can be achieved to inspire employees’ confidence in the organization (Tabor et al., 2019). Thirdly, selfless love is to establish the value of mutual care in the organizational culture, and show care and respect for employees, so that employees feel understood and appreciated, and then produce a sense of integrity, harmony and happiness in the organization (Bayighomog and Arasli, 2022).

There are two types of benefits that spiritual leadership can bring when it is embedded in the workplace, and they are at individual and organizational level (Fry, 2003). The former mainly brings spiritual development or growth to individuals, such as increasing personal happiness and achievement, creativity, increasing personal honesty and trust, and improving organizational commitment. The latter is to improve organizational harmony, enhance organizational performance and bring long-term success to enterprises (Butts and Rokhsar, 1999). Spiritual leadership nourishes the organizational commitment of employees. Attaches great importance to the spirit will help employees away from the negative emotions, attaches great importance to the personal meaning, personal values and life purpose, etc., can cause employees to inner vision and goals, so employees can feel the value its self, and have a sense of being understood and appreciated (Cho and Han, 2018).

Besides, spiritual leadership show a positive impact on employee job satisfaction, productivity, organizational competitive advantage and performance. The leader’s caring behavior for employees will make them emotionally attached to the organization and then willing to stay in the organization (Settoon et al., 1996; Yang et al., 2019b). The behavior presented by spiritual leaders will first affect the psychological perception of employees, then affect the commitment of employees to the organization, and also drive the improvement of performance. Spiritual leadership can not only help employees understand the meaning of work, but also promote more positive interactions with colleagues. When employees can accept the values of the group to which they work, they like and are willing to engage in work (Fry and Kriger, 2009). So we put forward the first hypothesis:

Hypothesis 1. Spiritual leadership is positively associated with employee job engagement.

### The Mediating Role of Emotion Regulation

Spiritual leaders may influence individuals’ behavior and emotion *via* emotive means (Kahn, 1990). One of the key criteria qualifying an individual to be a spiritual manager is the implementation of policies and emotive behaviors that align with his/her emotive principles. Emotion regulation, defined as a kind of process and ability of controlling and regulating one’s own emotion when facing matters, can affect employees‘ behaviors in organization and is regarded as an important strategy to manage the behavioral outcomes.

Individuals can regulate the tone and kinetic energy of emotions. The former is a variety of basic emotions, such as happiness, sadness, anger, sadness, etc. The latter is the intensity, duration and recovery time of emotions (Jiao and Lee, 2021). Early emotional research toward emotional tone, focuses on the individual’s state of mind, explore the emotional structure and its forming reasons, researchers pay attention to the different emotional facial expressions and the emergence of emotional state and individuals correction (Fry et al., 2005), which can reflect the emotions aroused, which is an important argument of emotion function theory for emotion regulation.

Emotion regulation focuses on how to deal with the emotions aroused, which can not only alleviate the negative emotion, but also maintain the positive emotion (Gross and John, 2003), so that individuals can achieve goals and effectively adapt to the workplace when facing the internal and external requirements. Emotional arousal is a kind of motivation for individuals to achieve their goals (Gross, 2013). However, whether emotions can help individuals achieve their desires and produce healthy adaptive behaviors depends on whether individuals can correctly evaluate the purpose of emotions and the types of emotional responses when they are aroused by emotions. This process of correct evaluation influences the relationship between emotion and behavior, which is called emotion regulation. In addition to repairing unhappy emotions, emotion regulation can also help to maintain a good emotion when facing difficulties. When individuals continue to maintain positive psychological state and face potential threats, they can view them with a more optimistic and relaxed attitude, and are better able to adapt to the threat brought by practice than those in a continuous negative emotion, and are less prone to pressure (Gross and Ochsner, 2008).

When an individual is in a certain aroused emotional state, the individual can self-perceive the emotion, and try to adjust the emotion by means of behavioral and cognitive changes, so as to achieve the purpose of adapting to the environment, and the individual expects and believes that the adjustment is effective (Ali et al., 2020). Emotion regulation is the process of attempting to change the original emotional state when people change their cognition and behavior by assessing the needs of the situation, thus affecting emotional events, emotional intensity, emotional duration or emotional expression (Gross and Munoz, 1995; Gross and Thompson, 2007).

Emotion regulation is a situationally sensitive and can change emotions according to different situations to meet current needs or achieve desired goals (Gross, 1999). Therefore, this study draws on the emotional regulation strategies of cognitive reappraisal and expressive suppression proposed by Gross and John (2003). Cognitive reappraisal is a kind of cognitive change, which involves explaining a potential emotional withdrawal situation in a non-emotional way, thus changing the impact of the current emotion. Cognitive reappraisal tries to understand the negative emotions such as frustration, anger and disgust in a more positive way, or rationalize the emotional events. It is an antecedent-focused emotion regulation strategy (Gross and John, 2003). On the contrary, Gross (2013) pointed out that expressive suppression is a type of response, and takes place in the process to restrain ongoing emotional expressions.

As shown above, spiritual leaders can help employees overcome destructive emotions such as fear, anger, sense of failure and pride, thus improving employees’ feelings of happiness and organizational performance. In the communication with employees, leaders’ selfless love makes employees feel cared and respected, and makes them more integrated into their work roles, thus improving their work performance (Schaufeli and Bakker, 2004; Daft, 2008). Spiritual leadership leads to closer integration of individuals and groups within an organization and greater alignment of values and goals (Jiao and Lee, 2021). If leaders can make good use of spiritual leadership, they can not only help employees to improve their spiritual level, but also strengthen their centripetal force and work engagement, and make their work performance more stable, thus encouraging their emotion reappraisal.

Emotional regulation is also correlated with individual social support. The results show that cognitive reappraisal is more likely to express positive and negative emotions and be accepted by colleagues (Gross, 2013). On the contrary, expression suppressors tend to inhibit positive and negative emotional expression. Expression suppressors resist sharing their emotions and tend to be pessimistic and passive, which affects their social networks and depletes individual social support. Cognitive re-evaluators, on the other hand, express more positive emotions, view things positively, have better interactions and connections with others, and have better social functions (Gross and John, 2003; Gross, 2013).

A large number of studies have found that cognitive reappraisal and expressive suppression have different effects on emotion, cognition and social behavior (Gross and John, 2003). Emotion regulation can not only slow down negative mood, but also maintain positive mood, so that individuals can achieve goals and effectively adapt to society when facing internal and external requirements or fear (Gross, 2013). Therefore, emotion regulation can not only repair unhappy mood, but also maintain a good emotion. When individuals maintain positive psychological state, they are more optimistic and relaxed when facing potential threat events.

Job engagement means engagement, commitment, enthusiasm, focus and vitality, including attitude and behavior. The antecedents of such attitudes and behaviors lie in the circumstances under which employees work, and then affect the values and effectiveness of the organization. According to role theory, job engagement refers to the degree to which individuals are engaged in their work and immersed in their roles (Yuan et al., 2015). Personal sense of purpose and concentration show initiative, adaptability, effort and persistence to organizational goals, that is, employees show unconditional efforts at work and synchronously put cognition, emotion and physiology into individual work roles (Eisenberg et al., 2000; Wang M. et al., 2019), thus enhancing their daily role play in the workplace. Spiritual leaders can pay attention to employees’ career development and daily life, regulate reasonable salary system and choose retention talent strategy, which will increase employees’ centripetal force to the organization, thus reducing turnover rate and promoting job engagement (Zou et al., 2020). In other words, as long as the organization gives appropriate conditions, employees will show their due role performance. Employees with a high degree of dedication and contribution will perform better at work and be confident in their work.

Cognitive reappraisal, on the other hand, can lead employees to change their cognition and they will be more willing to pay for work with whole heart, even when encounter difficulties and problems, they still can stick to it. It is easier to for employees to reduce the depletion of the mood with cognitive reappraisal (Srivastava et al., 2006; Shin and Hur, 2020), at the same time, the exuberant energy and good emotion can be of help for employees to accept the challenge and enthusiasm for work. Under these circumstances, employees will not easily get job burnout (Zou et al., 2020). In addition, when employees are in a stable and happy state, they will fully focus on work and have high job engagement without burning out. Besides, if employees prefer the strategy of cognitive reappraisal to regulate their emotions, they will use the form of reinterpretation to regulate their psychology, so that the difficulty will turn for the better. They can persist in the work even when they encounter difficulties or bottlenecks, have high enthusiasm and are willing to engage in the work, and feel the importance of work to them, which can help them improve job engagement and role immersion (Gross and John, 2003; Schaufeli and Bakker, 2004).

When in the heavy workload and under time pressure, expressive suppression can weaken the subjective feeling, and be more prone to emotional exhaustion, which cause employees not easy to gain self-esteem and achievement and make them unable to produce a sense of belonging in the organization (Zhou and Mou, 2021), thus reducing the role immersion. Therefore, they tend to show a negative and indifferent attitude at work, which leads to psychological problems and a strong sense of job burnout, thus affecting job engagement. When confronted with pressure, if employees take the strategy of expressive suppression, neither to adjust the mentality, nor let out the negative emotion, they will experience more negative emotions, and not be able to concentrate on inputs to the work, and gradually accumulate depression and negative pessimistic psychological feeling (Coat et al., 1996), which can lower the job satisfaction and achievement, thus reducing job engagement.

Therefore, we hypothesize the following:

Hypothesis 2. Cognitive reappraisal mediates the positive relationship between spiritual leadership and employee job engagement.
Hypothesis 3. Expressive suppression mediates the positive relationship between spiritual leadership and employee job engagement.

**FIGURE 1:**
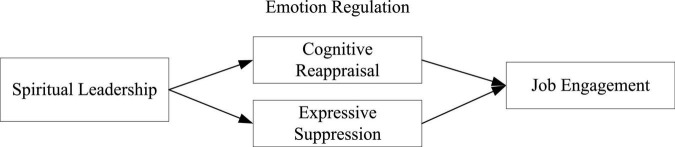
Theoretical Model.

Figure 1 depicts our conceptual model.

## Materials and Methods

### Sample and Procedures

Full-time employees at eighteen high-tech firms located in Pearl River Delta region in China were invited to participate in our survey. Author contacted the human resources department of the firms to introduce the research project and asked the director of HR for survey help. The firms agreed to participate in the survey, and all participants believed that this survey is very interesting and agreed to finish the survey. Author promises confidentiality of their responses to all respondents.

The surveys were collected during employees’ lunch time. In order to reduce common method bias, we collected data at different times. First, focal employees reported on their perceptions of their immediate supervisor’s spiritual leadership, their own emotion regulation, and the control variables at T1 (time 1). Second, after 1 month, we invited respondents in T2 (time 2) to rate their own job engagement behavior.

The data collection process lasted 4 months, firstly we distributed 557 surveys, 330 completed surveys at T1. After checking the original 330 surveys, all samples are valid at this stage. Then 330 respondents answered the surveys at T2, and 203 surveys are useful for the research after deleting 127 item missing surveys, with a valid rate of 36.4%. The majority of the participants were male (73.8%), with an average age of 36.31 years (SD = 4.93) and average tenure of 4.97 years (SD = 2.04). We evaluated non-response bias (203 vs 127) by testing for differences in means for each variable (*t* > 0.05) and compared the former and latter questionnaires (106 vs 97) and found that non-response bias (*t* > 0.05) doesn’t exist.

### Measurement

Because the items in the questionnaire were originally developed in English. We translated them into Chinese by strictly following the back-translation procedures (Brislin, 1986). All the items in this study were rated on a five point Likert-type scale (1 = strongly disagree, 5 = strongly agree). Supplementary Appendix A presents the measurement items we used in our study.

#### Spiritual Leadership

We measured spiritual leadership using Fry et al.’s (2005) seventeen-item scale. The Cronbach’s alpha for this measure was 0.896. Vision was measured using Fry et al.’s (2005) six-item scale. The Cronbach’s alpha was 0.884. Hope and Faith was measured using Fry et al.’s (2005) five-item scale. The Cronbach’s alpha was 0.755. Selfless love was measured using Fry et al.’s (2005) six-item scale. The Cronbach’s alpha was 0.885.

#### Emotion Regulation

We measured cognitive reappraisal using Gross and John’s (2003) six-item scale. The Cronbach’s alpha for this measure was 0.874. Expressive suppression was measured using Gross and John’s (2003) four-item scale. The Cronbach’s alpha was 0.794. The Cronbach’s alpha for motion regulation was 0.794.

#### Job Engagement

We measured job engagement using Schaufeli et al.’s (2002) fourteen-item scale. The Cronbach’s alpha for this measure was 0.821. Vigor was measured using Schaufeli et al.’s (2002) five-item scale. The Cronbach’s alpha was 0.864. Dedication was measured using Schaufeli et al.’s (2002) five-item scale. The Cronbach’s alpha was 0.854. Absorption was measured using Schaufeli et al.’s (2002) four-item scale. The Cronbach’s alpha was 0.827.

#### Control Variables

According to previous studies (e.g., Chen et al., 2014), we controlled for employees’ gender, age, and tenure, because these factors may influence participants’ emotion and engagement in workplace (Benefiel, 2005). Prior research show that education level is acrucial factor facilitating emotion control (Chen et al., 2019).

### Hypotheses Testing

In path analysis, structural equation modeling (SEM) is considered as a better way by using AMOS6.0, which allows simultaneous estimation of multiple indirect paths and provides specific model fit indices (James et al., 2006). We first tested our hypothesized measurement model and compared different models (base model and other alternative models). Then we tested the hypotheses to make sure if they were supported. Finally, in order to test the mediation effect of emotion regulation further, we use bootstrapping to check hypotheses 2 and 3.

Table 1 shows the correlation matrix. We can see from Table 1 that job engagement is significantly related to spiritual leadership (*r* = 0.16, *p* < 0.05), cognitive reappraisal (*r* = 0.27, *p* < 0.01), and expressive suppression (*r* = −0.19, *p* < 0.05) all being positively related to employee job engagement, respectively.

**TABLE 1:** Correlation matrix (*N* = 203).

Variables	1	2	3	4	5	6	7	8
1. Age	1							
2. Gender	–0.07	1						
3. Tenure	–0.02	0.09	1					
4. Education level	0.03	–0.01	0.16*	1				
5. Spiritual leadership	–0.06	0.01	0.06	0.04	1			
6. Cognitive reappraisal	0.02	–0.09	–0.10	0.07	0.24**	1		
7. Expressive suppression	–0.05	–0.02	0.06	–0.14	−0.30***	0.13	1	
8. Job engagement	–0.02	–0.09	0.12	0.17*	0.16*	0.27**	−0.19*	1
Mean	39.18	0.69	5.54	2.25	3.62	3.64	3.44	3.59
S.D.	7.94	0.46	2.81	0.65	0.72	0.95	0.94	0.65

*p < 0.05; **p < 0.01; ***p < 0.001.

To assess the construct validity of the variables, we conducted confirmatory factor analyses (CFA) in Table 2. The results showed that the hypothesized four-factor model fit the data best (χ2 = 269.24, df = 135, χ2/df = 1.99, CFI = 0.94, GFI = 0.98, TFI = 0.92, RMSEA = 0.05).

**TABLE 2:** Results of confirmatory factor analyses (*N* = 203).

Variable	χ 2	df	CFI	GFI	TFI	RMSEA
Hypothesized Model (four-factor model)	269.24	135	0.94	0.98	0.92	0.05
Three-factor model (cognitive reappraisal and expressive suppression combined into one factor)	393.64	139	0.90	0.84	0.83	0.09
Two-factor model (cognitive reappraisal, expressive suppression, and spiritual leadership combined into one factor)	535.27	142	0.81	0.78	0.80	0.11
One-factor model	719.84	145	0.74	0.70	0.71	0.13

Although our data were collected at different period of time, all variables (i.e., spiritual leadership, cognitive reappraisal, expressive suppression, and job engagement) were collected from the same source, thus causing common method biases. In order to examine if common method variance can affect our results, we firstly conducted a Harman’s one-factor test to make sure whether the first variable can explain most of the variance, and we found that the first factor can only explain 32.45% of variance. Then we included the 41 items collected from the same employee into one model and compared its model fit indices with the measurement model. We found that the one-factor model had a poor fit with the data set (χ2 = 532.67.92, df = 136, χ2/df = 3.92, CFI = 0.80, GFI = 0.74, TFI = 0.76, RMSEA = 0.14). Hence, we believe that common method bias cannot be a concern in this study.

**FIGURE 2:**
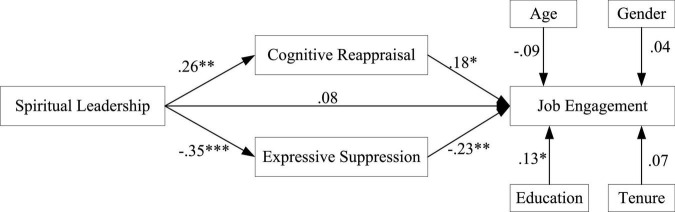
Results of path analysis. *p < 0.05; ^**^p < 0.01; ^***^p < 0.001.

After evaluating the factor construct and common method bias, we then test all hypotheses by using SEM. As predicted in hypothesis, there is a positive relationship between spiritual leadership and employee job engagement. Empirical analysis was conducted to examine this relationship as shown in Figure 2. The results of SEM show that some control variables has no significant impact on job engagement (gender: β = 0.04, *null*; age: β = −0.09, *null*; tenure: age: β = 0.07, *null*) while education level has significant effect on job engagement (β = 0.13, *p < 0.05*). As shown in Figure 2, all path coefficients supported the hypotheses. Specifically, first, spiritual leadership was positively related to cognitive reappraisal (β = 0.26, *p* < 0.01) and negatively to expressive suppression (β = −0.35, *p* < 0.001). Further, cognitive reappraisal was positively related to job engagement (β = 0.18, *p* < 0.05) while expressive suppression was negatively related to job engagement (β = −0.23, *p* < 0.01). In addition, with the presence of emotion regulation, i.e., cognitive reappraisal and expressive suppression, the path between spiritual leadership and employee job engagement became insignificant (β = 0.08, *null*), indicating cognitive reappraisal and expressive suppression play a full mediation effect between spiritual leadership and job engagement, respectively. The above results provided initial support for Hypotheses 2 and 3.

To further and directly examine our proposed mediation effects, we performed bootstrapping procedures using Hayes bootstrapping techniques (Preacher and Hayes, 2008). With 10,000 iterations in model 4, we found that the indirect effect of spiritual leadership on employee job engagement through cognitive reappraisal was 0.07, with a 95% bias-corrected bootstrap confidence interval of (0.0049, 0.1344), which does not contain zero, thus supporting the mediating effect in Hypothesis 2. Further, the same process was exerted to test the mediating role of expressive suppression. The indirect effect of spiritual leadership on employee job engagement *via* expressive suppression was −0.08, with a 95% bias-corrected bootstrap confidence interval of (−0.0359, −0.0007), again not containing zero, thus providing support for Hypothesis 3. In all, according to the results of SEM and bootstrapping, all hypotheses are supported by the data.

## Discussion and Implications

Our study is the pioneer to examine the effects of spiritual leadership on employees’ job engagement in workplace. Based on emotion regulation and leadership theories, this study testified the influencing mechanism of spiritual leadership on employees’ job engagement. Specifically, our findings indicate that spiritual leadership is one of crucial antecedents of employees’ job engagement. Furthermore, both cognitive reappraisal and expressive suppression were found to be the mediating factors that account for the impact of spiritual leadership on job engagement. Our findings have important theoretical and managerial implications.

### Theoretical Contributions

First, our research uses an emotive lens to examine the role of spiritual leadership in shaping employees’ job engagement behavior. Although prior studies have found that spiritual leadership is significantly related to job satisfaction and organizational citizenship behavior at employee level and firm growth at organizational level (e.g., Posner et al., 2005; Cho and Han, 2018), its effect on employees’ job engagement gained little attention. Our findings demonstrate that spiritual leadership is essential for job engagement, which can be regarded as a kind of emotive relevance. Future research can take an emotive view to find other valuable leadership styles to facilitate the job engagement.

Second, while most extant studies on spiritual leadership rely upon theoretical perspectives such as leader-member exchange (e.g., Schaufeli and Bakker, 2004; Jiao and Lee, 2021) to explain the impact of spiritual leadership on employee behavior, we employ role theory to integrate emotion regulation and leadership to examine the effects of spiritual leadership on job engagement. Specifically, the mediating mechanism of cognitive reappraisal and expressive suppression can explain more of the impact of spiritual leadership on job engagement and role immersion from the view of emotion regulation, which can enhance our understanding of the role of spiritual leadership in organizations. One positive path and the other negative path can provide us with a special explanation on job engagement when employees face emotional crisis.

Third, our results indicate that cognitive reappraisal and expressive suppression are equally important for motivating job engagement. This result further reinforces the long-held view of spiritual leadership as an emotive style because it can exert selfless love and respect to employees, which can strengthen the positive emotion and change the negative emotion into positive one or reduce the bad effects of emotions (Wang L. et al., 2019). Opposite to the previous study (e.g., Dent et al., 2005; Hunsaker, 2020), expressive suppression, though having negative effects on job engagement, can be transformed or relieved by using spiritual leadership. In short, our findings to a large extent support the effectiveness of the dual opposite path influence carried out by spiritual leadership in shaping followers’ emotion and behavior.

### Practical Implications

Our study provides important insights into how leaders facilitate employees’ job engagement by using spiritual leadership. First, this study argues that spiritual leaders play an important role in facilitating job engagement for better performance. To increase job engagement at workplace, managers may show an effective and influential vision to keep employees together as a driving force motivate employees. Besides, managers can set challenging goals for employees and encourage them to succeed in their work. Most importantly, managers should be patient with employees, especially when they are in trouble or negative emotions. Leaders should be honest and not hypocritical and can stand up for their employees. Speaking of emotion regulation, employees should learn how to control and express their emotions in time and in right ways. Cognitive reappraisal can be a crucial way to deal with emotions to enhance job engagement. On the contrary, expressive suppression can be alleviated by using the right leadership style. Only in this way can employees put their heart in their work. In a word, employees can make use of cognitive reappraisal to enhance their positive emotion and reduce the harmful effect of expressive suppression when facing negative emotions.

### Limitations and Future Directions

When interpreting the contributions, we know that there are some limitations in this study. First of all, this research focused on emotion regulation as a mediating factor. But we know that there are many kinds of emotion regulation strategies, such as situation selection, situation modification and attentional deployment and they can influence the relationship between leadership style and job engagement, for example, attentional deployment may influence the emotive behavior and attract emotion from the current negative mood, thus causing positive effect on job engagement (Eisenberg et al., 2000). Future research can focus on the effects of the above emotion regulation strategies which might depict a quite different picture in accounting for the variance in employee job engagement. Most importantly, the different functions exerted by different emotion regulation strategies can be compared to find the differentiated mechanism on the relationship between spiritual leadership and job engagement, which will be better to facilitate the usage of pragmatic emotion regulation strategies.

Another limitation is that our study uses a cross-sectional data to test the hypotheses, the direction of causality cannot be fully articulated. Emotion regulation and job engagement changes over time, so does leadership style, which may cause some changes among their relationships in the long run. We argue in this study that emotion regulation can mediate spiritual leadership and job engagement, but it maybe alternative for the above relationship. Future research should focus on the longitudinal data and test the causality over time.

Finally, generalizability of the results is limited because the data comes from one economic zone, which is one of the most developed area in China. Therefore, researchers should make surveys in other zones, developed areas such as Beijing-tianjin area and Yangtze River Delta area and underdeveloped such as the northeast and the west areas. Because different areas show differentiated features in regional culture, firm culture and management styles, especially different development level may cause different leadership and emotive cognition, which may influence the generalizability of the results in this research. Most importantly, the comparison of the results from different areas can show us some insights in the near future.

## Data Availability Statement

The raw data supporting the conclusions of this article will be made available by the authors, without undue reservation.

## Author Contributions

YH did author work independently, including conception design, data collection, and result analysis during the writing process.

## Conflict of Interest

The author declares that the research was conducted in the absence of any commercial or financial relationships that could be construed as a potential conflict of interest.

## Publisher’s Note

All claims expressed in this article are solely those of the authors and do not necessarily represent those of their affiliated organizations, or those of the publisher, the editors and the reviewers. Any product that may be evaluated in this article, or claim that may be made by its manufacturer, is not guaranteed or endorsed by the publisher.
