# Supplementary material for: Spiritual Leadership and Job Engagement: The Mediating Role of Emotion Regulation
Source: Front Psychol. 2022 Apr 14;13:844991. doi: 10.3389/fpsyg.2022.844991 (PMC9046577; doi:10.3389/fpsyg.2022.844991)
Supplement: Supplementary file 1 [file Table_1.docx]

**Appendix：Supplementary Material--Measurement items of variables**

**Job engagement scale (revised from Schaufeli *et al*., 2002)**

Vigor

1. I feel mentally strong when I’m working.

2. I can work for a very long time.

3. When I work, I feel like I am full of energy.

4. When working I feel energetic.

5. After getting up in the morning, I am eager to go to work.

Dedication

1. I find my work to be full of meaning and purpose.

2. I am inspired by my work.

3. I am enthusiastic about my work.

4. I am proud of my work.

5. I find my work challenging.

Absorption

1. Time flies when I’m working.

2. When I am working, I forget everything else around me.

3. I feel happy when I am working intensively.

4. I can get carried away by my work.

**Emotion regulation measurement items (Gross and John, 2003)**

Cognitive reappraisal

1. I control my emotions by changing the way I think about the situation I'm in.

2. 2. When I want to feel less negative emotion, I change the way I'm thinking about the situation.

3. When I want to feel more positive emotion, I change the way I'm thinking about the situation.

4. When I want to feel more positive emotion (such as joy or amusement), I change what I'm thinking about.

5. When I want to feel less negative emotion (such as sadness or anger), I change what I'm thinking about.

6. When I'm faced with a stressful situation, I make myself think about it in a way that helps me stay calm.

Expressive suppression

1. I control my emotions by not expressing them.

2. When I am feeling negative emotions, I make sure not to express them.

3. I keep my emotions to myself.

4. When I am feeling positive emotions, I am careful not to express them.

**Spiritual leadership (Fry *et al*., 2005)**

Vision

1. I understand the company's vision and am willing to commit myself.

2. The vision of my work group can stimulate employees' potential.

3. The vision of my company can motivate me to perform well.

4. I have faith in the vision of the company.

5. The vision of our company is clear and makes me look forward to it.

6. I have confidence in the company and am willing to do my best to ensure the success of the mission of the company.

Hope and confidence

1. I am willing to persevere and make efforts to help the company succeed, because I have confidence in the philosophy of the company.

2. I always do my best at work because I have faith in the company and its leaders.

3. To show my confidence in the company, I will try my best to achieve the mission to help the company succeed.

4. I set challenging goals for my work because I believe the company wants me to succeed.

5. My company really cares about its employees.

Selfless love

1. My company is very kind and considerate to its employees

2. When employees encounter difficulties, the company is willing to help and solve the problem.

3. In my company, leaders practice what they preach.

4. My company treats its employees sincerely and sincerely.

5. My company doesn't punish people who own up to mistakes.

6. Leaders in my company stand up for their employees.
